# Protocol for mindfulness-oriented recovery enhancement (MORE) in the management of lumbosacral radiculopathy/radiculitis symptoms: A randomized controlled trial

**DOI:** 10.1016/j.conctc.2022.100962

**Published:** 2022-07-03

**Authors:** Ryan S. Wexler, Devon J. Fox, Hannah Edmond, Johnny Lemau, Danielle ZuZero, Melissa Bollen, Diane Montenegro, Anand Parikshak, Austin R. Thompson, Nels L. Carlson, Hans L. Carlson, Anna E. Wentz, Ryan Bradley, Douglas A. Hanes, Heather Zwickey, Courtney K. Pickworth

**Affiliations:** a Helfgott Research Institute, National University of Natural Medicine, Portland, OR, USA; b Department of Orthopaedics and Rehabilitation, Oregon Health & Science University, Portland, OR, USA; c Herbert Wertheim School of Public Health and Human Longevity Sciences, University of California, San Diego, La Jolla, CA, USA

**Keywords:** Mindfulness, Lumbosacral radiculopathy, Low back pain, RCT protocol, Virtual intervention, Mindfulness-oriented recovery enhancement

## Abstract

**Introduction:**

Lumbosacral radiculopathy/radiculitis (LR) or “sciatica” is a commonly intractable sequelae of chronic low back pain (LBP), and challenges in the treatment of LR indicate that persistent pain may have both mechanical and neuropathic origins. Mindfulness-based interventions have been demonstrated to be effective tools in mitigating self-reported pain in LBP patients. This paper describes the protocol for a randomized controlled trial (RCT) evaluating the effects of the specific mindfulness-based intervention Mindfulness-Oriented Recovery Enhancement (MORE) on LR symptoms and sequelae, including mental health and physical function.

**Methods:**

Participants recruited from the Portland, OR area are screened before completing a baseline visit that includes a series of self-report questionnaires and surface electromyography (sEMG) of the lower extremity. Upon enrollment, participants are randomly assigned to the MORE (experimental) group or treatment as usual (control) group for 8 weeks. Self-reported assessments and sEMG studies are repeated after the intervention is complete for pre/post-intervention comparisons. The outcome measures evaluate self-reported pain, physical function, quality of life, depression symptoms, trait mindfulness, and reinterpretation of pain, with surface electromyography (sEMG) findings evaluating objective physical function in patients with LR. To our knowledge, this is the first trial to date using an objective measure, sEMG, to evaluate the effects of a mindfulness-based intervention on LR symptoms.

**Hypotheses:**

We hypothesize that MORE will be effective in improving self-reported pain, physical function, quality of life, depression symptoms, mindfulness, and reinterpretation of pain scores after 8 weeks of mindfulness training as compared to treatment as usual. Additionally, we hypothesize that individuals in the MORE group with abnormal sEMG findings at baseline will have improved sEMG findings at their 8-week follow-up visit.

## 1 Introduction

### 1.1 Background and rationale

Low back pain (LBP) is a ubiquitous musculoskeletal complaint that often presents as a chronic, intractable condition with an estimated lifetime prevalence of 85.5% [1,2] and global point prevalence of 8.2% [3]. Lumbosacral radiculopathy/radiculitis (LR), also known as “sciatica”, is a secondary neuropathic condition to LBP, affecting approximately 37% of this population [4]. LR is most frequently caused by compression of the L4-S1 nerve roots, subsequently causing pain, sensory loss, motor/reflex abnormalities, and weakness [5,6].

Diagnosis of LR is completed using electrodiagnostic studies, magnetic resonance imaging, and/or physical examination. Surface electromyography (sEMG), a type of electrodiagnostic study, has been found to be an effective tool in assessing LR based on its ability to differentiate between compressive and non-compressive radiculopathies [4,7] as well as its strong diagnostic accuracy (93.6%) for differentiating between the most common distal spinal disc herniations, L5 and S1 [8,9]. sEMG assesses muscle tone and function on the lower legs while walking. LR is characterized by pain and dysfunction in the lower extremity; thus, evaluating muscle function between symptomatic and asymptomatic sides provides clear objective data regarding physical function. Additionally, sEMG has been used to quantify the severity of impairment in motor function and thus may help elucidate relationships between objective functional data and self-reported pain scores.

In addition to being pervasive, treatment for LR patients is financially burdensome. The U.S. estimated annual costs of spinal fusions in 2015 was $10 billion, and indirect costs of general chronic pain has been estimated to total $100 billion annually [10,11]. These indirect costs comprise the most financially burdensome element of neuropathic pain management [12]. Mental health burdens associated with LBP are also significant [1,6], with studies reporting rates of depression up to 72% in samples of chronic pain patients [13]—suggesting that treatment of mood symptoms in chronic pain patients may decrease both health care costs and pain-related dysfunction [14,15]. Prescription pain management, including opioid medications, is common in this population due to otherwise ineffective and expensive treatment options, resulting in 19% of these patients eventually becoming long-term users of opiates [16].An estimated 29% of chronic pain patients will misuse and 11% will abuse opiates, underscoring the need for alternative treatments for LBP patients that do not have associated risk for dependence, abuse, and addiction [17].

The burdens associated with LR warrant a more cost-effective and safe pain management strategy. Research has shown mindfulness-based interventions (MBIs) are more cost-effective than usual care [18] and effective in limiting self-reported pain in patients with chronic LBP, neuropathic conditions, and depression [[19], [20], [21], [22], [23], [24], [25], [26], [27], [28]]. Potential mechanisms for the relationship between mindfulness practice and self-reported pain include decreases in reactivity of immune and supraspinal pathways within and between the amygdala [29], pro-inflammatory cytokines [30], thalamus, and periaqueductal gray matter [31]. Though MBIs have been effective in other pain management contexts, including generalized LBP, sufficient demonstrative evidence of the efficacy of MBIs for pain management in LR specifically is lacking.

Mindfulness-Oriented Recovery Enhancement (MORE) was developed for the treatment of addiction, stress, and pain using cognitive-behavioral therapy and positive psychology to help disrupt attentional bias towards pain in chronic pain conditions [[32], [33], [34], [35], [36], [37],^39^]. It differs from other MBIs such as mindfulness-based stress reduction in its focus on the development of reappraisal and savoring practices. These appear throughout the program as key tenets of the pain restructuring process and are able to achieve reductions in pain perception by shifting participants’ attention from affective processing to sensory processing and reducing experiences of anhedonia by regulating negative emotional states [38]. Dr. Garland, the developer of MORE, proposes a model “leveraging [three] natural rewards:” savoring natural rewards, noticing and generating pleasant internal states, and cultivating meaning and self-transcendence [39]. When MORE was evaluated for its 9-month impact on chronic pain and opioid use against a supportive psychotherapy program, 45% of MORE participants ceased misusing opioids after 9 months, and 50% of MORE participants saw a minimally clinically important change in pain severity. This is compared to 24.4% in opioid misuse cessation and 29.3% for pain severity changes in the supportive psychotherapy group [40]. Although various MBIs have been investigated for their effects on LBP [[19], [20], [21], [22], [23], [24], [25],^36^,^41^], few studies have specifically evaluated the effectiveness of MBIs for LR symptoms, and none have attempted to deliver MORE virtually in this subpopulation. To our knowledge, this is also the first trial to date using an objective measure, sEMG, to evaluate the effects of a MBI on LR symptoms.

## 2 Trial design

This manuscript outlines the protocol for a randomized clinical trial to evaluate the efficacy of a virtually administered, 8-week MORE intervention on self-reported pain, physical function, quality of life (QoL), depression symptoms, trait mindfulness, and sEMG findings in patients with LR when compared with treatment as usual (TAU).

## 3 Methods

### 3.1 Study setting

IRB approval was received for this protocol at the National University of Natural Medicine (NUNM), and study recruitment began in February 2021 (IRB #: KP112720; clinicaltrials.gov #: NCT04818606). Recruitment is conducted through flyers at the NUNM academic and clinic buildings as well as clinical partnerships with the Oregon Health & Science University Spine Center and Comprehensive Pain Center, Northwest Integrative Medicine, and other local primary care clinics. Additionally, flyers are posted in local grocery stores and community newsletters. Clinical partnerships consist of a 2-part recruitment strategy: 1) query of patients via clinic electronic medical records using ICD-10 code, age, and being English-speaking as search criteria; or 2) direct referral from physicians to our study e-mail and phone number. Once contact information for eligible patients is received via queries with partner clinics, recruitment letters are sent. Two weeks later, study staff reach out via phone to conduct screenings and schedule baseline visits with potential participants.

Participants undergo a telephone screening and interested participants who meet all eligibility criteria are invited to schedule a baseline visit at NUNM's Helfgott Research Institute building in Portland, OR. Participants without a previous LR diagnosis (Table 1, inclusion criterion 1b) may be eligible to schedule a baseline study visit if meeting a minimum pain score of 15 on the modified painDETECT Questionnaire (PD-Q). Eligibility criteria are summarized in Table 1.

**Table 1:** Eligibility criteria.

Inclusion Criteria	Exclusion Criteria
1a) Presence of lumbosacral radiculopathy/radiculitis symptoms that extend below the knee secondary to low back pain for greater than 6 weeks with a painDETECT score greater than 15 OR	1) Have received epidural steroid injection in the prior 3 months
1b) Diagnosis of lumbosacral radiculopathy/radiculitis secondary to low back pain that extends below the knee, with symptoms present for greater than 6 weeks with the following ICD-10 codes: M54.16, M54.17, M51.16, M51.17, M47.26, M47.27, M54.40, M54.41, M54.42, M99.53, M99.54, S34.21, S34.22, G54.4, and G55	2) Inability to complete 20 unassisted gait cycles
2) At least 18 years of age and not older than 65 at the time of study enrollment.	3) Have received a surgical intervention for low back pain or lumbosacral radiculopathy/radiculitis within the previous 6 months
3) Ability to read and understand English	4) Current active mindfulness meditation practice: 1 time/week or more and/or formal training in mindfulness/meditation practice
4) Willingness to be randomized to either an experimental or a control group	5) Concurrent diagnosis of cancer
5) Willingness to refrain from unnecessary or self-directed pain management/treatment plan changes during study enrollment and to report necessary changes made	6) Allergy or intolerance to adhesive
6) Daily access to the internet via cell phone, tablet, or computer	7) Current unmanaged or uncontrolled mental illness known to cause psychosis: schizophrenia and schizotypal disorders, bipolar I disorder with psychosis, major depressive disorder with psychosis

### 3.2 Study design

Baseline visits conducted at Helfgott Research Institute include confirmation of eligibility criteria, informed consent, study questionnaires, and sEMG collection for 20 gait cycles. Follow-up visits include all steps except repeated informed consent. All participants receive an email containing the informed consent document after baseline visit. Within 16 weeks of baseline visit completion, participants are randomized to either an 8-week MBI and treatment as usual (MBI + TAU) or TAU-only group to be completed in parallel cohorts. Both groups are asked to refrain from changes to their current treatment plan whenever reasonable (i.e., to avoid self-directed changes in treatment such as starting a new supplement) and to report any medically necessary (i.e., physician-recommended or -implemented) changes made during study enrollment. All study participants receive a copy of a pain education handout, “Understanding Pain,” at the time of intervention initiation as an effort to reduce the disparity upon health education [42]. This handout is provided publicly by the Oregon Pain Management Commission. Participants in both groups are asked to report daily pain levels using a Visual Analog Scale (VAS) administered via text message or email as preferred per participant.

**Fig. 1:**
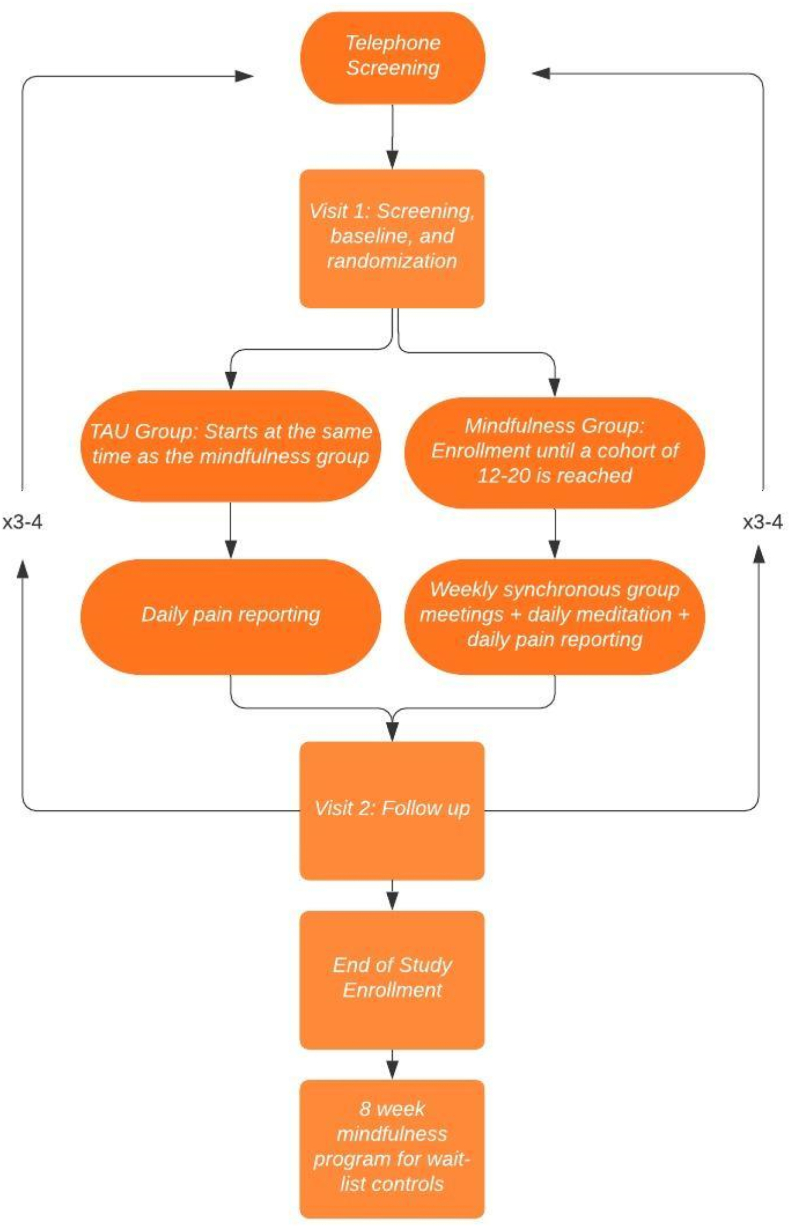
Overview of study design.

Treatment as usual was chosen as the control condition for this experiment, as experts recommend using TAU control groups in studies of MBIs based on evidence from other RCTs conducted in this realm [43]. MORE has not been studied in the context of LR, and no gold standard has been identified as an active control for MORE in this subpopulation; therefore, TAU was deemed as appropriate for this study design. The control group undergoes TAU over 8 weeks of parallel group comparison and starts their 8-week enrollment period on the same day as the experimental group. Participants assigned to the TAU control group are offered to join a MORE program be delivered after the data collection period of the trial has ended. Our study team offered this option as we anticipate a benefit from MORE in the management of lumbosacral radiculopathy. A study flowchart can be seen in Fig. 1.

### 3.3 Randomization and blinding

Cohorts of 24–46 (12–23 per group) participants are recruited into the study and undergo group assignment via simple randomization using a random number generator. Enrollment concludes at their follow-up visit within 6 weeks after the end of their 8-week reporting (intervention) period. To conserve scientific integrity and minimize the introduction of researcher bias, research assistants conducting study visits are blinded to participant group assignment (single-blind). Allocation concealment is maintained by limiting access to the random sequence until the moment of assignment, and a new random sequence is generated for each cohort. Randomization assignment is completed by the study coordinator using a list of anonymized study IDs for that cohort. To prevent experimenter bias, research staff providing the intervention will not be allowed to run follow-up study visits for participants in any cohort to which they have delivered an intervention. Participants unable or unwilling to complete an in-person follow-up visit are provided with the option to complete the questionnaires virtually; this policy was implemented to optimize retention given the ongoing global pandemic and anticipated challenges with in-person follow-up visits.

### 3.4 Intervention

Participants randomized to the intervention group undergo an 8-week mindfulness training program as instructed by the “MORE for the Treatment of Chronic Pain” manual. The intervention consists of weekly virtual sessions during which participants are asked to follow along with a guided meditation read by a trained MORE instructor and to engage in reflection and discussion exercises with other study participants. MORE instructors are trained via a two-day intensive workshop, the only education in MORE available at the time of this publication. The planned MORE instructor for this trial has delivered other mindfulness interventions such as Being Present 2.0 [44] and is certified in delivery of Cultivating Emotional Balance. All sessions are conducted using the virtual conference platform Zoom®; session topics are outlined in Table 2. The main techniques of MORE, mindfulness, reappraisal, and savoring, are delivered in session 1, 3, and 4. Mindfulness within MORE prompts participant awareness of pain oriented attention; reappraisal asks participants to “consider new ways of thinking about a stressful situation; ” and savoring encourages participants to direct attention towards rewarding experiences [45].

**Table 2:** MORE session topics.

Session 1	What is Pain and Why Can Mindfulness Help?
Session 2	Automaticity in Chronic Pain
Session 3	Mindful Reappraisal
Session 4	Mindful Savoring
Session 5	Relationship between Pain and Unhealthy Coping Habits
Session 6	The Stress Response to Pain
Session 7	Pain and Thought Suppression
Session 8	Review and Discussion of Maintaining a Mindfulness Practice

To maintain anonymity, participants chose a “screen name” at their baseline study visit that is associated with them at session log in and participants were encouraged, not required, to participate in group discussion using their camera and microphone. Participants in the intervention group complete weekly activities and daily guided mindfulness practices via pre-recorded MP3 files at home consistent with the MORE protocol [34]. Audio from the synchronous sessions is recorded and stored securely for participants absent from synchronous sessions. Participants who do not attend sessions without providing advance notice to the study team will be contacted using the combination of communication methods to which they indicated they preferred at the baseline visit (i.e. phone, text, or e-mail). Attempts to contact the participant will be made until the participant has reached the maximum allowed number of missed sessions, at which point they will be declared lost to follow up. In addition to completing a daily VAS and reporting treatment changes, MORE participants are also asked to report their daily time spent practicing mindfulness. All adverse events occurring during the study, including those observed by or reported to the research team, will be recorded.

### 3.5 Harms

Adverse events (expected), unexpected adverse events (UAE), and serious adverse events will all be described using a detailed documentation form and logged in the Research Electronic Data Capture (REDCap) [46,47] system to be summarized and reported to the institutional IRB and with future publications. Expected adverse events described in consent documentation include mental health exacerbations or triggers associated with meditation and/or completing self-report questions as well as scrapes or allergic reactions associated with sEMG procedures.

### 3.6 Outcome measures

Instruments used include a battery of self-reported questionnaires for demographics, health history, pain, physical function, quality of life (QoL), depression, and mindfulness; an additional and objective measure of physical function is sEMG recording collected baseline and follow-up visits. Questionnaires are completed using the REDCap online data management platform. MBI + TAU participants are asked to complete a daily practice log with their VAS to track their mindfulness practice in minutes.

*Demographic and personal contact Questionnaire:* In addition to common demographic characteristics, this questionnaire asks participants to report their primary clinic for treatment of LR. Participants are also asked to provide the screen name they are using to log into video conferencing for synchronous sessions.

*Lumbosacral Radiculopathy/Radiculitis Health History Questionnaire:* This questionnaire, developed by the study team, collects information on the duration of patient's symptoms, current and previous treatments including duration, and the perceived effectiveness of previously attempted treatments.

*Modified Oswestry Low Back Pain Disability Questionnaire/Oswestry Disability Index (ODI):* The ODI is designed to assess the intensity of pain and the degree to which pain interferes with activities of daily living such as personal care, lifting, walking, sitting, etc. The ODI has been found to have high sensitivity, specificity, validity, and test-retest reliability for patients with low back pain [[48], [49], [50], [51], [52], [53]].

*Visual Analog Scale (VAS)*: The VAS is a self-reporting scale where participants are asked to report their pain on a scale from 0 to 10 where 0 represents “no pain” and 10 represents “worst pain.” The VAS has been used in many pain intervention studies and has shown to have high reliability, validity, specificity, and sensitivity [51,54,55]. The VAS has established validity and reliability and has been used to establish a minimum clinically important difference and a minimum detectable change for other pain questionnaires [56,57].

*painDETECT Questionnaire (PD-Q):* The PD-Q is a self-reporting pain questionnaire that is designed to assess the presence of neuropathic pain in patients with chronic low back pain and lumbosacral radiculopathy/radiculitis. The PD-Q is scored on a scale from −1 to 38 where scores 19 or greater indicate likely presence of neuropathic pain, scores from 12 to 18 represent ambiguous pain, and scores below 12 represent a likelihood that neuropathic pain is not present. The PD-Q has been found to have high sensitivity, specificity, validity, and test-retest reliability in patients with low back pain and lumbosacral radiculopathy/radiculitis [4,[58], [59], [60], [61], [62]].

*SF-12 Patient Questionnaire for Quality of Life (SF-12 QoL):* The SF-12 QoL assesses an individual's overall quality of life using self-reporting questions to determine an individual's ability to accomplish and complete activities of daily living as well as their overall mood and outlook on life [63]. The SF-12 has been found to have acceptable reliability and validity and has been recommended as a useful alternative to the SF-36 if questionnaire length is a concern [51,[63], [64], [65]].

*Major Depression Inventory (MDI):* The MDI is a self-reporting questionnaire that includes questions about depression symptoms consistent with the *DSM-V* guidelines for major depressive disorder. The MDI is commonly used in research to determine the degree to which an individual is currently experiencing a depressed mood and has shown to have high sensitivity, specificity, validity, and test-retest reliability for patients with low back pain [66,67]. Participants who score above a 30, indicating severe depression symptoms, will be sent a referral for medical management.

*Five Facet Mindfulness Questionnaire (FFMQ):* The FFMQ is a self-reporting questionnaire that assesses an individual's trait mindfulness using five “facets” or categories of mindfulness: Observing, Describing, Acting with Awareness, Nonjudging of inner experience, and Nonreactivity to inner experience. Participants respond to 39 questions such as, “I can easily put my beliefs, opinions, and expectations into words”, on a scale of never or very rarely true (1) to very often or always true (5). The FFMQ has demonstrated good construct, discriminant, and predictive validity, as well as its internal consistency and correlation with negative affective symptoms [[68], [69], [70], [71]].

*Mindful Reinterpretation of Pain Sensations (MRPS) scale:* The MRPS is a 9-item survey that asks participants to rate their level of agreement with statements such as “I try to watch my pain from a distance, as if I were an objective observer,” and “I “zoom in” close to the pain to see what sensations it is made up of” on a scale ranging from, “never do that”, (0) to, “always do that”. (6). The MRPS scale was designed by Dr. Eric Garland, the developer of MORE, based on the reinterpretation component of the Coping Strategies Questionnaire [72]. The MRPS scale is currently pending validity publications but is preliminarily reported to have high sensitivity to the effects of MORE.

**Fig. 2:**
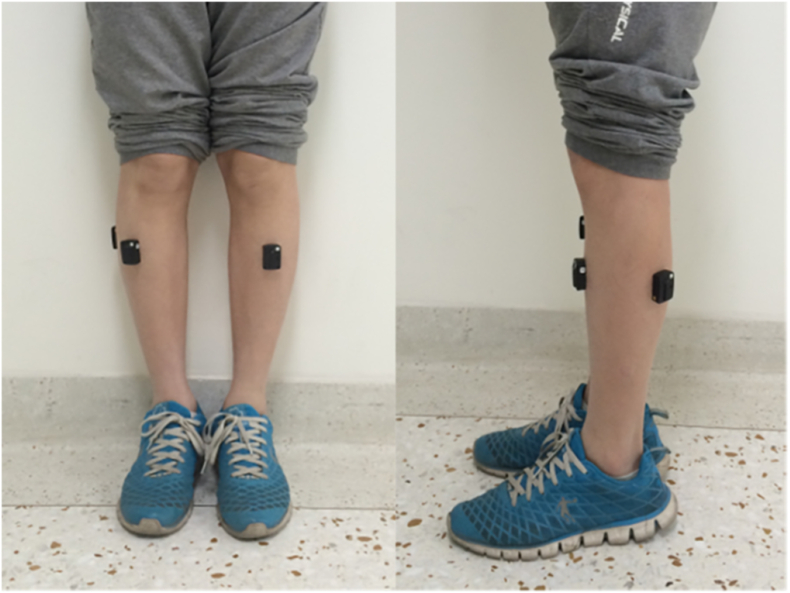
sEMG Sensor and Electrode Placement [73].

*Surface Electromyography (sEMG):* Participants undergo sEMG to evaluate the function of the anterior tibialis and lateral gastrocnemius muscles, which have been shown to be dysfunctional in patients with L5 and S1 nerve root compression [8,9,73]. For administration of sEMG, study personnel utilize Thought Technologies sEMG Equipment: ProComp Infiniti System with BioGraph Infiniti Software (Model #T7500 M), EMG MyoScan-Z Sensor (Model#: T9503Z, 2048 samples/second), EMG Myoscan-Pro Sensor (Model#: T9401M-60, 256 samples/second), and the Tele-Infinit Compact Flash (Model#: T9600) for wireless communication of sEMG data. The MyoScan-Z Sensors are be placed on the lateral gastrocnemius of both legs, as this muscle is more commonly affected in LR and a valuable target for high sampling rate diagnostic equipment. Root mean square (RMS), a component derived from an sEMG wave, represents the force/torque produced by muscles [74], measured in microvolts, and correlates with weaker contractions in the presence of chronic pain. [[75], [76], [77], [78]], while RMS peak time represents the time during the gait cycle at which RMS was highest. These two variables will be used for analysis of physical function as previously studied [9]. Fig. 2 depicts the placement of sEMG electrodes.

### 3.7 Power and sample size calculations

Using results of Braden et al., a standard deviation of 8.3 points for the Oswestry Disability Index was used for effectiveness of mindfulness-based stress reduction in LBP patients [79]. An effect size of Cohen's *d* = 0.83 was calculated when using the reported minimal clinically important difference of a 10-point change [57,80]. Effect sizes ranging from 0.5 to 1.1 have been found previously for mindfulness interventions using outcome measures of pain and physical function in mechanically and neurologically derived LBP. The large effect sizes powered for in this study are considered acceptable due to the large effect sizes found in studies using MORE with multiple outcomes measures [35,36,41]. Using the GPower program (version 3.1.9.4) for *a priori t*-test sample size calculations using *alpha* = .05 yields 80% power to detect an effect of treatment of size *d* = 0.83, with a sample size of 48 participants. When adjusting for an expected retention rate of 80%, a total proposed sample size of 60 participants is required (*n* = 30 per group).

### 3.8 Statistical analytical plan

Participant descriptive information collected at baseline, including demographics, pain characteristics, physical function, QoL, depression, and trait mindfulness will be reported as mean (SD) or number (percent), as appropriate, for each study group. Likewise, changes from baseline to follow-up of each outcome measure will be reported as mean (SD) for each group.

*A priori* analytical modeling has been developed based on primary and secondary outcome measures and established relevant covariates. Relevant covariates (according to priority) include age, sex [7], change in treatment during study enrollment (as a binary variable), disease etiology [81,82], duration of symptoms prior to study enrollment, previous condition specific surgery [81], and baseline instrument score. To retain adequate power for linear mixed modeling, only covariates that are correlated to the outcome measure of interest (*r* ≥ .3, by Pearson or point-biserial correlation, as appropriate) will be retained. Covariates found to be significantly correlated will be added to the relevant model in a stepwise fashion in the aforementioned order.

All outcome variables aside from VAS pain (pain severity, physical function, QoL, depression symptoms, trait mindfulness, and reinterpretation of pain) will be analyzed using linear mixed modeling with maximum likelihood estimation of missing data to compare mean change between the MBI + TAU and TAU groups’. Next, we will evaluate associations between pain, physical function, QoL, depression symptoms, trait mindfulness, and reinterpretation of pain in relation to time spent practicing mindfulness amongst participants in the intervention group. The same approach will evaluate for significant differences in RMS and RMS-peak time within sEMG findings from baseline to follow-up. Finally, we will evaluate the association between changes in RMS from baseline to follow-up and change scores of the ODI, VAS, and PD-Q in participants in the intervention group.

For all models, we will complete a modified intention-to-treat analysis (mITT) including all those participants who completed the program. A per-protocol structure using only those participants in the treatment group who completed a minimum of 5 sessions synchronously will be used for sensitivity analysis as has been used previously in clinical trials and trials of MORE [41,83,84].

Evaluation of daily VAS scores will be conducted using growth curve analysis with day 1 of the intervention for the respective cohort as the intercept to account for changes in VAS occurring between baseline and intervention initiation.

## 4 Discussion

It has been previously shown that mindfulness based interventions are an efficacious treatment option for patients with chronic low back pain and other chronic musculoskeletal conditions [[19], [20], [21], [22], [23],^25^]. While LBP patients have been thoroughly studied in MBIs, adults experiencing ongoing LR symptoms may have a unique symptomology that is best treated with a multimodal approach to pain management [14,19]. Many conventional strategies for long-term management of radicular pain are expensive and partially effective [85], highlighting the importance of exploration into alternative pain management options. Because chronic pain conditions often involve a bidirectional relationship between physical and psychological symptoms and states, MORE represents a potentially efficacious treatment protocol for patients with LR than has previously been studied and described [35,36].

Strengths of this study design are its thorough eligibility criteria for recruitment of LR patients as well as its novel design. To date, this is the only study evaluating an MBI for which LR is a condition for eligibility and randomization, whereas previous research has only performed stratified analyses, which are subject to methodological limitations. This study is novel in its use of MORE for LR patients and in its virtually-delivered MORE intervention strategy. A second strength is in using sEMG to determine its sensitivity to change pre/post-intervention for LR and its association with self-reported pain and physical function. This helps to expand the limited literature in this specific sEMG application. A third strength of this study is its virtual delivery as the virtual design allows for a significant reduction in the number of potential contagion exposures and infection risk amidst the COVID-19 pandemic. The virtual design also increases accessibility of MBIs for patients in rural areas and for those with limited transportation or severe disability. Lastly, this study design is able to capture both self-reported and objective outcome measures.

This study is limited in its participant diversity. As not all study questionnaires are available or validated in other languages, this study does not allow for the recruitment of non-English speaking participants. Similarly, Portland, OR is a majority white metropolitan area and racial diversity is limited. A second limitation is the varying sampling rate of the sEMG sensors used. This study was designed to use available equipment at the Helfgott Research Institute; ideally, all sensors used would have a minimum sampling rate of 1024 samples/second and be capable of collecting power spectrum data such as median and mean power frequency [86]. Challenges of this study reside in the evaluation of the data due to the novel nature of the study. Aspects of this study such as sEMG effectiveness in pre/post-intervention have limited literature and may present unique challenges in analysis and data collection. COVID-19 may also present additional challenges in recruitment with participants unwilling to adhere to COVID-19 guidelines or uncomfortable coming into a public space for their baseline and follow-up visits. Lastly, the large cohort sizes used in this study, 12–23 participants per group, are greater than the reported optimal group sizes for active psychotherapy interventions, 6–15 [38,[87], [88], [89], [90]]. Financial and time limitations associated with the study setting lead to need for a condensed recruitment timeline that favored fewer, larger cohorts compared with the optimal scenario of more cohorts that were smaller in group size. Future studies will likely benefit from optimal group/cohort sizing to maximize therapeutic benefit.

This study explores a distinct MBI that has not been previously investigated in this format nor with this specific subpopulation, it and responds to the existing need for more research in the effectiveness of MBI subclasses. Results gleaned from this RCT will provide valuable information regarding the relationship between chronic pain and mindfulness within the LR subpopulation. Finally, this novel area of research will provide direction for both future academic and clinical pursuits among patients experiencing chronic neuropathic pain.

## Declaration of competing interest

The authors declare that they have no known competing financial interests or personal relationships that could have appeared to influence the work reported in this paper.
